# University Staff and Students’ Attitudes towards a Completely Smoke-Free Campus: Shifting Social Norms and Organisational Culture for Health Promotion

**DOI:** 10.3390/ijerph18137104

**Published:** 2021-07-02

**Authors:** Marguerite C. Sendall, Lauren Fox, Darren Wraith

**Affiliations:** Faculty of Health, School of Public Health and Social Work, Queensland University of Technology, Brisbane, QLD 4059, Australia; lauren.fox@icon.team (L.F.); d.wraith@qut.edu.au (D.W.)

**Keywords:** cigarette smoking, tobacco control, smoke-free policy, smoking attitudes, university setting, health promotion, public health, organizational culture

## Abstract

A large university in Queensland, Australia with a diverse staff and student community introduced a campus wide smoke-free policy in 2016. The purpose of this enquiry was to understand attitudes about a new smoke-free policy, its potential impact and the shift in social norms and organizational culture to inform the next phase of implementation. An electronic survey was distributed to all staff and students approximately 12 weeks after the smoke-free policy was implemented. The survey consisted of multiple-choice questions about demographics, smoking behaviour, attitudes towards smoking and tobacco control, awareness of the smoke-free policy, and attitudes towards the effect of a completely smoke-free campus on quality of life, learning and enrolment. The survey was completed by 641 university staff and students. Respondents reported seeking out (80.4%) and socialising in smoke-free environments (86.6%) and supported smoke-free buildings (96.1%), indoor areas (91.6%), and outdoor areas (79%). The results revealed overwhelming support for a completely smoke-free campus (83%) and minority support for designated smoking areas (31%). Overall, respondents reflected positively towards a campus wide smoke-free policy. These findings suggest Queensland’s early adoption of tobacco control laws influenced the social environment, de-normalised smoking, changed behaviour, preference for smoke-free environments and shifted social norms. These findings provide convincing evidence for organisational change and suggest health promotion policy makers should progress the implementation of smoke-free policies nationally across the higher education sector.

## 1. Introduction

Tobacco smoking is the leading cause of preventable death and disease in Australia, contributing to more than 1 in 8 deaths or almost 21,000 lives in 2015 [1]. The negative health effects of smoking and exposure to second-hand tobacco smoke are well documented in scientific literature in Australia and internationally [2]. In 2005, the social and economic cost of tobacco surpassed AUD (Australian dollars) 31 billion [3]. However, in 2015–2016, the social cost of tobacco use was estimated at nearly AUD 137 billion [4]. While the jump in estimated costs may be explained by different estimation measurements, most of the difference is due to dependent smokers spending on tobacco, workplace costs and premature mortality causing a reduction in economic output, pain and suffering attributed to poor health and premature mortality, and the value of life lost [4].

In the early 1980s, evidence emerged linking lung cancer in non-smokers with exposure to second-hand smoke [5]. This evidence shifted tobacco smoking from an individual health problem to a wide-scale environmental public health issue which required regulatory action [6]. Since then, the Australian Commonwealth, State and Territory Governments have employed a comprehensive and coordinated approach towards tobacco control [7]. Implementation of strategies including smoke-free legislation, advertising bans, plain packaging, price increases and anti-smoking campaigns in Australia has resulted in a steady decline in smoking prevalence and the social acceptability of tobacco use [8]. In 1991, 24% of Australians aged 14 years and older reporting daily smoking compared to 12% in 2016 [9]. The Tobacco Working Group of the Preventative Health Taskforce believe if smoking prevalence is less than 10% in 2020, tobacco smoking rates will continue to decline until rates are so low it would no longer be one of Australia’s most important health issues [9]. A substantial reduction in the number of adolescent and young adults taking up smoking will be required to achieve this goal.

Modifying the environment through the introduction of smoke-free legislation is an effective health promotion strategy for tobacco control. Sound evidence-based smoke-free policy underpinned by environmental change and supported by effective implementation, leads to behaviour change and a shift in social norms and organisational culture in settings such as university campuses. Australia has a long and successful history of smoke-free policies, with the first smoking restrictions commencing in the 1970s [10]. Australia’s toughest anti-smoking laws commenced in September 2006, when the Queensland government banned smoking in all restaurants, bars, clubs, workplaces, and commercial dining areas. Following this, smoking prohibitions were implemented at all schools, hospitals, healthcare and residential aged-care facilities, and public transport waiting points. Smoking is also banned within 10 m of patrolled beaches, campsites and public amenities, children’s playgrounds, and under-18 sporting events, and within five meters of any non-residential building. In 2016, the Australia Medical Association and the Australian Council on Smoking and Health recognised Queensland as the nation’s leader in tobacco control and health promotion efforts. Modifying the smoking environment in public places has directly influenced health behaviour and contributed to a broader cultural shift towards smoking. Effectively, Queensland set the precedence for environmental change enacted through smoke-free policy, provided a framework for population behaviour change, and significantly and effectively shifted the culture of tobacco use. Since 1998, the number of daily smokers in Queensland has more than halved [11]. 

The introduction of smoke-free policies has coincided with a reduction in reported respiratory problems, improved cardiovascular health and lung function among previously exposed non-smokers [12]. These policies act as an environmental framework to influence behaviour and positively impact the health of current smokers by reducing the number of cigarettes smoked per day, supporting cessation and decreasing smoking rates [13]. Smoke-free policy adoption has modified the environment and played a significant role in shifting the public’s perception of smoking and social and cultural attitudes. International research suggests there is a growing global preference for smoke-free environments. This shift in social norms and societal culture predicts increasing support for and compliance with modifying environments through smoke-free policies [13].

Studies conducted in the United States confirm smoke-free policies positively impact smoking behaviour and exposure to second-hand smoke on university campuses [14,15]. From 2012 to 2017, the number of US college and campuses which adopted smoke-free or tobacco-free policies more than doubled. In 2012, 774 campuses completely prohibited smoking (smoke-free) or smokeless tobacco use and combustible tobacco product smoking (tobacco free) in all indoor and outdoor areas. By 2017, 84% of 2082 college and university campuses were tobacco-free [16]. However, there is a paucity of evidence about staff and students’ attitudes about the smoke-free policy and potential health promotion impact and how this shift in organisational culture informs the next phase on policy implementation within Australian context. The shift in societal attitudes is evidenced by previous research which shows Australians, including people who currently smoke, are supportive of smoke-free policies [10,17]. Other university-based studies found smoking behaviours among students to be grounded in organizational culture and opportunistic and predictable based on apparent social norms, exposure to peer smoking behaviour and accessibility to designated smoking areas [15]. Environmental change underpinning smoke-free policy as a behaviour change mechanism and a shift in organisational culture should consider the contextualised like-nuances of settings such as university campuses. As a result of this changing landscape and shift in social norms, societal attitudes and organisational culture and policy implementation, 32% of universities in New Zealand, 25% in the United States and 5% in the UK have introduced smoke-free policies [18,19]. 

By 2016, twenty-two out of forty public universities in Australia had implemented smoke-free policies [20]. The remaining eighteen public universities allowed smoking in designated areas on campus or had other restrictions in place. In Queensland, only two universities had introduced campus wide smoke-free policies. These policies replicated related prohibitions outlined in The Tobacco and Other Smoking Products Act 1998 [21]. University campuses remained notably excluded from smoke-free educational facilities outlined in The Tobacco Act. From 1 July 2018, all public universities in Queensland implemented total smoke-free policies. 

Prior to 1 July 2016, smoking was allowed in designated areas on campus in the University under investigation. From January 2016, the University communicated information about the new policy through several channels such as broadcast emails, the website, local forums and meetings and signage. The University allowed a twelve-month transition period. Transition strategies included advertising about implementation and operationalisation of the smoke free policy, marketing about smoking cessation resources including counselling, clear and regular communication signage, re-design of designated smoking areas, and staff training and education sessions. The new policy prohibits smoking in all areas of the university campus, including the grounds, buildings and vehicles. It includes the use of cigarettes and all other tobacco-related products including herbal cigarettes, loose tobacco, cigarette-making equipment and electronic cigarettes [22]. 

The purpose of this study was to understand attitudes about the potential impact of a smoke-free policy and the shift in social norms and organizational culture to inform the next, institutionalisation phase at a large public university in Queensland, Australia.

## 2. Materials and Methods

### 2.1. Setting

The setting for this study is a large public university in Queensland, Australia. The university has over 50,000 student enrolments—more than 38,000 are fulltime and nearly 9000 are international. The university employs over 4700 staff on two city campuses with a total revenue of $1.06 billion [23]. Student and staff characteristics at QUT are reasonably representative of most large universities in Australia, although some of the largest universities have higher international student enrolments. 

### 2.2. Participants

Following the implementation of the campus wide smoke-free policy in July 2016, an electronic survey was distributed to all current staff and students. An invitation to participate in this survey was included in the Registrars newsletter emailed during September 2016. The newsletter provided a short introduction about the research and a link to the survey. A follow-up email was sent in October 2016. The survey consisted of multiple-choice questions about demographics, smoking behaviour, attitudes towards smoking, tobacco control, awareness of the smoke-free policy, and attitudes towards a completely smoke-free campus on quality of life, learning and enrolment.

### 2.3. Data Collection

The survey used to collect data was developed and validated by researchers at a university in Western Australia [24]. The questions were from previously validated instruments [25], validated for content validity by eight health promotion, research and tobacco experts and a test–retest (*n* = 32) was used to measure reliability. The survey was anonymous and consisted of several multiple-choice questions. 

Respondents were grouped by their reported smoking status. Statement questions were developed to measure respondent’s attitudes towards smoking and the new smoking restrictions and indicated their level of agreement to statements using a five-point interval Likert-type scale, for example, strongly disagree/disagree/neutral/agree/strongly agree. Respondents were asked if they are aware of any [university] policy which restricts smoking on campus. A follow up question asked respondents to describe the university’s current smoking policy. Lastly, respondents were asked their opinion about the impact a smoke-free policy would have on staff and student quality of life, student learning, and student enrolment.

### 2.4. Data Analysis

The data were analysed using SPSS for Windows 10 and R [26]. Descriptive analysis of the data presents an overview of respondents’ characteristics. Chi-square analysis was applied to determine the views and attitudes towards a campus wide smoke-free policy for current smokers, ex-smokers and non-smokers. For efficiency of reporting and as the main objective was to ascertain broad levels of agreement with the statements, strongly agree and agree were combined (and similarly strongly disagree and disagree). As the sample respondent characteristics differed from characteristics in the QUT population, responses were post-stratified using sex, primary role and international student status to assign survey weights (See Table 1: Respondent demographics.). For post-stratification, the ‘survey’ package in R was used [27]. 

## 3. Results

Overall, 641 university staff and students completed the survey. The demographic representations of survey respondents and known demographics at QUT are described in Table 1.

### 3.1. Attitudes towards Smoking 

Less than half of the respondents (*n* = 252, 45.8%) were exposed to second-hand smoke on campus. Of these, only 15.4% reported exposure to second-hand smoke once or more in a day, 25.5% reported exposure to second-hand smoke once or more than once a week, and 24.7% reported exposure to second-hand smoke a few times a month. A significant difference was observed in the level of exposure between current smokers, non-smokers and ex-smokers (*p* = 0.002). 

Most respondents (*n* = 548, 86%) agreed with the statement “If someone smokes cigarettes around me, they are causing me harm”. A significant difference was observed in responses from current smokers, non-smokers and ex-smoker (*p* < 0.001), with 38.1% of current smokers agreeing with the statement. See Table 2: Agreement with smoking attitude statements reported by university staff and students.

### 3.2. Attitudes towards Tobacco Control

The overall attitude towards smoking was negative. Most respondents agreed they would “Prefer to socialise in a smoke free environment” (*n* = 553, 86.8%), “Rather date a non-smoker” (*n* = 543, 85.4%), “Seek out smoke-free environments” (*n* = 510, 80.4%), and were “Disappointed when a friend who does not normally smoke, smokes cigarettes while drinking alcohol” (*n* = 412, 64.9%). In responses between current smokers, non-smokers and ex-smokers, significant differences were observed (*p* < 0.001) for all four statements. When asking others to not smoke around them, current smokers were less likely (3.2%) to agree with the statement (*p* < 0.001), compared to non-smokers (55.6%) or ex-smokers (25.8%).

With reference to tobacco control attitude statements, most respondents agreed the “Campus should be smoke-free in all buildings” (*n* = 610, 96.1%), “All indoor worksites should be smoke-free, including bars and restaurants” (*n* = 579, 91.6%), “The campus should be completely smoke-free” (*n* = 528, 83%) and “The campus should be smoke-free including all outdoor areas” (*n* = 494, 79%). Significant differences were observed between responses from current smokers, non-smokers and ex-smokers, (*p* < 0.001) for all statements, with current smokers less likely to agree than non-smokers or ex-smokers. See Table 3: Agreement with tobacco control attitude statement reported by university staff and students.

### 3.3. Awareness of Smoke-Free Policy

Regarding awareness about any [university] policy restrictions on smoking, most respondents knew about the new smoke-free policy (*n* = 582, 91.4%). Very few were unaware or “did not know” (*n* = 55, 8.6%). No significant difference in responses between current smokers, non-smokers and ex-smokers was observed (*p* = 0.639). When asked to describe the current policy, most respondents affirmed “staff, students and visitors are banned from smoking tobacco throughout the campus; this includes all university buildings, grounds and vehicles” (*n* = 559, 84.3%). No significant differences was observed in responses from current smokers, non-smokers and ex-smokers (*p* = 0.217).

### 3.4. Attitudes towards a Completely Smoke-Free Campus Policy

Most respondents agreed a smoke-free policy would have a positive effect on staff quality of life (*n* = 522, 82.1%) and students’ quality of life (*n* = 536, 84.5%). Over half of respondents (*n* = 403, 63.5%) felt the policy would have a positive effect on student learning. Less than half (*n* = 305, 48.2%) felt the policy would have a positive impact on student enrolment. Significant differences were observed between responses from smokers, non-smokers and ex-smokers (*p* < 0.001) for all statements, with smokers less likely to acknowledge positive effects of the policy. See Table 4: Agreement with effects of a completely smoke free campus policy statement reported by university staff and students.

For successful implementation, respondents were asked about possible consequences for non-compliance with the smoke-free policy. Most respondents (*n* = 501, 78.6%) agreed with a verbal reminder. Half (*n* = 322, 50.5%) agreed with a monetary fine. Approximately one-third suggested anti-smoking education (*n* = 234, 36.7) or disciplinary processes for non-compliance (*n* = 218, 34.2%). Less than a quarter (*n* = 140, 22%) felt community service should be one of the consequences. Very few respondents (*n* = 34, 6.8%) felt there should be no consequence for non-compliance with the policy.

## 4. Discussion

This research offers valuable insight into staff and students’ attitudes towards the newly implemented smoke-free policy, its impact and a shift in social norms and organisational culture at a large university in Queensland, Australia. Findings showed smoking prevalence among respondents to be relatively low (6.5%) compared to current smoking rates in Australia (12%) [8] and Queensland (11%) [28]. Attitudes towards smoking were mostly negative, reflecting an organisational shift in social norms and corresponding with the general negative attitudes towards smoking in Australia [29]. These attitudes and shift in organisational culture maybe viewed as pro-tobacco cessation. The data showed significant support for the smoke-free policy as the majority of respondents (including current, ex and non-smokers) were likely to seek out smoke-free environments (80.4%) and preferred to socialise in smoke-free environment (86.6%). Most respondents agreed the campus should be smoke-free in all outdoor areas (79%), all buildings (96.1%), and indoor worksites including bars and restaurants (91.6%). Overall, these social norms and the organisational culture reflect a broader shift in societal attitudes in Queensland through the enactment of sequential and progressive smoke free legalisation.

These results imply a compounding social, cultural and environmental effect stemming from the roll-out of smoking bans across Queensland for more than a decade and supports behaviour modification, social norming and organisational change through policy based environmental change. Especially relevant are policies modifying environments by prohibiting smoking at schools, restaurants, bars, workplaces, commercial eating and drinking areas and other public spaces such as public parks and beaches. This substantial shift in tobacco-related norms was confirmed by the majority preference towards a completely smoke-free environment (83%) and little support for preserving designated smoking areas (31%). These results correspond with the increasing denormalization and decreasing prevalence of smoking in Queensland. Many respondents agreed the policy would have a positive impact on students’ quality of life (84.5%), staff quality of life (82.1%) and student learning (63.5%). Overall, respondents reacted favourably to a complete smoking ban compared to those in Western Australia who responded less favourably in most questions [24]. These findings suggested the gradual and consistent approach towards modifying the environment and addressing behaviour change through tobacco control in Queensland has influenced public preference, social attitudes, health behaviour change and support for smoke-free environments.

The introduction of smoke-free policies in workplaces, restaurants and bars has been critical in reshaping the public perception of smoking, smoking behaviour and the demoralisation of tobacco in Queensland. In this instance, denormalization refers to the transition in the status of smoking from a socially acceptable and widely practised behaviour to one increasingly characterised as destructive, unhealthy and anti-social [30]. Research confirms people who currently smoke have successfully adjusted to, accept and comply with smoke-free laws [31,32]. This suggests social norms and behaviour surrounding smoking have shifted to such a degree that current smokers accept restrictions without resistance. 

Increasing restrictions, about where smoking is permitted means people who smoke, are now being defined as a group whose behaviour entails segregation. Therefore, it is important for policymakers to consider how denormalization, social norms and organisational change affects those who continue to smoke within the context of policy-based environmental change. As smoking becomes increasingly concentrated among already disadvantaged populations (e.g., lower socioeconomic groups or those with mental illness), there is a risk these individuals and groups will feel even more marginalised and are less motivated to quit. Ongoing policy efforts to address the prevalence of tobacco smoking have reshaped the public perception of smoking as socially undesirable. This perception plays a major role in improving compliance with smoke-free policies by increasing feelings of dissonance or unease associated with nonconformity [33]. This may be especially relevant in a university setting given smoking is a socially constructed and maintained behaviour established in early adulthood [15]. 

Young people in Queensland experience a smoke-free environment from childcare, go to smoke-free schools and later, they will work in smoke-free workplaces. University campuses and other tertiary settings are an obvious omission in the smoke-free educational facilities outlined The Tobacco Act and are not covered by current tobacco laws. Research confirms individuals who quit smoking before the age of thirty almost eliminate the risk of mortality due to smoking-induced causes [34]. Thus, smoking prevention strategies focussed to young adults is vital to future tobacco control efforts in Queensland and Australia. 

As more young adults attend university and the age of smoking uptake increases, campuses provide an important setting for primary and secondary smoking prevention and cessation support [35]. These efforts should focus on young adults in the initiation phase of smoking who will undergo transition to either non-smoking or heavier smoking. University students are uniquely susceptible to peer-influence, social norms, and organisational culture during their transition to university life [36], may lose established support networks and feel pressured to ‘fit in’ with new social groups. These influences, coupled with unfamiliar pressures and external demands, may lead students to initiate compromised health behaviours which could persist throughout their lives [34]. According to the Queensland smoking prevention strategy 2017–2020, young adults aged 18–29 show the strongest decline in smoking rates in the last decade. This validates the reach and influence of tobacco control in Queensland and emphasises the need to double-up on these efforts [37]. By applying a consistent and focused approach towards tobacco control and introducing comprehensive anti-smoking laws reflecting an organisational shift in tertiary settings, Queensland will likely experience a further decline in smoking rates in coming years.

### Limitations

Firstly, the response rate was low in this population. This might be because the initial survey was distributed during a mid-semester break, or the link was embedded in an electronic newsletter. Secondly, there is potential bias due to the small sample size. Thirdly, there was a large proportion of non-smokers, females and domestic students who participated in this study. This may be due to selective non-response and underreporting from current smokers, males and international students. To adjust or correct for this imbalance, results were post-stratified to reflect the QUT population according to gender, primary role and international student status. However, there may be an imbalance between other groups and/or a possibility for respondents to differ in their attitudes to non-respondents. These issues apply more generally to survey results and are difficult to control. 

## 5. Conclusions

The results from this study suggest support for a campus wide smoke-free policy. These findings may reflect the influence of Queensland’s early adoption of environment modification though the implementation of successive tobacco control laws and a consistent approach towards normalising smoke-free environments on attitudes and behaviour towards smoking, social norming and shifted organisational culture. The legacy of early adoption may continue to have a significant impact on the social acceptability and denormalization of smoking in public spaces. Comprehensive tobacco regulation at Queensland universities may deter young adults from initiating smoking and offer cessation support for those current smokers who are contemplating behaviour change.

These findings have implications for the higher education sector because they contribute to the growing body of evidence for smoke-free policy implementation. These findings indicate a preference for smoke-free environments and a tolerance to smoke-free policies suggesting health promotion policy makers should progress the implementation of smoke-free policies nationally across the higher education sector. 

## Figures and Tables

**Table 1:** Respondent demographics.

		Sample, N (%)	Population (QUT), N (%)
Sex	Male	183 (28.5%)	25,334 (46.3%)
	Female	458 (74.5%)	29,735 (53.7%)
	TOTAL	641	54,709
Primary role	QUT Undergraduate student	272 (42.4%)	39,871 (70.3%)
	QUT Postgraduate student	94 (14.7%)	11,938 (21.1%)
	QUT General/professional staff member	206 (32.1%)	2627 (4.6%)
	QUT Academic staff member	69 (10.8%)	2259 (4.0%)
	TOTAL	641	56,695
International student status	Yes (international student)	93 (25.5%)	9769 (18.6%)
	No (domestic student)	273 (74.5%)	42,742 (81.4%)
	TOTAL	366 (100%)	100%

**Table 2:** Agreement with smoking attitude statements reported by university staff and students.

	Non-Smoker N (%) Adj.% (95% CI) *	Ex-Smoker N (%) Adj.% (95% CI) *	Current Smoker N (%) Adj. % (95% CI) *	Total N (%) Adj. % (95% CI) *	*p*-Value
If someone smokes around me they are causing me harm because of second hand smoke	456 (94%) 91.8% (88.5–95.2)	68 (76.4%) 68.9% (54.1–83.8)	24 (38.1%) 44.4% (28.9–60.0)	548 (86%) 83.3% (79.3–87.2)	<0.001
I prefer to socialize in a smoke free environment	469 (96.7%) 95.5% (92.9–98.1)	71 (79.8%) 70.2% (55.4–85.0)	13 (20.6%) 20.4% (8.2–32.7)	553 (86.8%) 83.2% (79.3–87.2)	<0.001
I seek out smoke free environments	441 (91.3%) 88.7% (84.9–92.4)	63 (71.6%) 70.1% (55.5–84.7)	6 (9.5%) 9.9% (0.0–19.2)	510 (80.4%) 76.6% (72.2–80.9)	<0.001
It disappoints me when a friend who does not normally smoke, smokes cigarettes while drinking	372 (77%) 75.7% (70.7–80.7)	36 (40.4%) 40.2% (25.6–54.7)	4 (6.3%) 4.0% (0.0–8.5)	412 (64.9%) 62.8% (57.8–67.7)	<0.001
I would rather date a non-smoker	458 (94.6%) 94.4% (91.9–96.8)	68 (76.4%) 67.2% (52.3–82.1)	17 (27%) 23.5% (10.8–36.2)	543 (85.4%) 82.4% (78.4–86.4)	<0.001
I ask others not to smoke around me	268 (55.6%) 51.1% (45.3–56.8)	23 (25.8%) 22.7% (10.6–34.7)	2 (3.2%) 1.8% (0.0–4.9)	293 (46.2%) 41.8% (36.9–46.7)	<0.001

Note: * refers to weighted or adjusted percentage estimates from post-stratification of survey results.

**Table 3:** Agreement with tobacco control attitude statement reported by university staff and students.

	Non-Smoker (N) % Adj. % (95% CI) *	Ex-Smoker (N) % Adj. % (95% CI) *	Current Smoker (N) % Adj. % (95% CI) *	Total (N) % Adj. % (95% CI) *	*p*-Value
Our campus should be smoke free including all outdoor areas	414 (87.9%) 85.6% (81.4–89.9)	64 (71.9%) 63.8% (48.8–78.8)	16 (25.4%) 26.7% (12.9–40.6)	494 (79%) 75.6% (71.1–80.1)	<0.001
The restrictions on where you can smoke makes it hard for smokers at the university	232 (48.3%) 49.2% (43.5–54.9)	43 (49.4%) 47.8% (32.6–63.1)	45 (71.4%) 66.4% (51.5–81.3)	320 (50.8%) 51.2% (46.2–56.2)	0.002
There should be some places at the university where people can go smoke	117 (24.4%) 25.1% (20.1–30.1)	32 (36%) 40.6% (25.5–55.7)	50 (82%) 81.5% (69.3–93.6)	199 (31%) 33.8% (29.0–38.7)	<0.001
There should be more help or support at the university for people who want to quit smoking	235 (48.7%) 53.8% (48.1–59.4)	39 (43.8%) 51.3% (36.1–66.4)	28 (45.2%) 39.9% (24.7–55.1)	302 (47.6%) 51.8% (46.8–56.9)	0.799
Our campus should be smoke free in all buildings	479 (99%) 98.9% (98.0–99.9)	81 (92%) 85.2% (72.7–97.8)	50 (79.4%) 77.6% (63.9–91.2)	610 (96.1%) 94.7% (92.2–97.2)	<0.001
Our campus should be completely smoke free	443 (91.5%) 89.9% (86.2–93.6)	70 (78.7%) 71.8% (57.2–86.4)	15 (23.8%) 26.2% (12.4–40.1)	528 (83%) 79.8% (75.5–84.0)	<0.001
All indoor worksites should be smoke-free, including bars and restaurants	463 (96.5%) 95.3% (92.6–97.9)	77 (86.5%) 75.8% (61.8–89.8)	39 (61.9%) 55.2% (39.8–70.7)	579 (91.6%) 88.0% (84.5–91.6)	<0.001

Note: * refers to weighted or adjusted percentage estimates from post-stratification of survey results.

**Table 4:** Agreement with effects of a completely smoke-free campus policy statement reported by university staff and students.

	Non-Smoker N (%) Adj. % (95% CI) *	Ex-Smoker N (%) Adj. % (95% CI) *	Current Smoker N (%) Adj. % (95% CI) *	Total N (%) Adj. % (95% CI) *	*p*-Value
Staff quality of life					
Negative	11 (2.3%) 2.7% (0.0–4.6)	8 (9%) 14.3% (1.9–26.8)	11 (17.5%) 19.4% (6.7–32.1)	30 (4.7%) 6.2% (3.6–8.9)	<0.001
Neither negative or positive	38 (7.9%) 9.7% (6.2–13.1)	12 (13.5%) 13.8% (3.1–24.4)	34 (54%) 45.6% (30.6–61.4)	84 (13.2%) 14.7% (11.1–18.4)	<0.001
Positive	435 (89.9%) 87.6% (83.4–91.4)	69 (77.5%) 71.9% (57.3–86.5)	18 (28.6%) 34.7% (19.7–49.6)	522 (82.1%) 79.1% (74.8–83.3)	<0.001
Student quality of life					
Negative	11 (2.3%) 2.7% (0.0–4.6)	7 (7.9%) 14.2% (1.7–26.6)	13 (20.6%) 25.3% (11.1–39.6)	31 (4.9%) 7.0% (4.2–9.9)	<0.001
Neither negative or positive	24 (5%) 5.6% (2.9–8.4)	12 (13.5%) 12.1% (1.9–22.2)	31 (49.2%) 38.5% (23.6–53.3)	67 (10.6%) 10.4% (7.0–13.6)	<0.001
Positive	447 (92.7%) 91.6% (88.4–94.9)	70 (78.7%) 73.8% (59.3–88.2)	19 (30.2%) 36.2% (21.2–51.2)	536 (84.5%) 82.5% (78.6–86.5)	<0.001
Student learning					
Negative	5 (1%) 1.1% (0.0–2.4)	5 (5.6%) 10.0% (0.0–20.7)	9 (14.3%) 18.3% (5.7–30.9)	19 (3%) 4.4% (2.1–6.7)	<0.001
Neither negative or positive	132 (27.3%) 25.4% (20.5–30.2)	39 (43.8%) 36.1% (21.4–50.8)	42 (66.7%) 59.9% (44.6–75.1)	213 (33.5%) 30.9% (26.3–35.4)	<0.001
Positive	346 (71.6%) 73.5% (68.6–78.4)	45 (50.6%) 53.9% (38.6–69.1)	12 (19%) 21.8% (8.8–34.8)	403 (63.5%) 64.8% (60.0–69.5)	<0.001
Student enrolment					
Negative	15 (3.1%) 2.9% (1.0–4.8)	5 (5.6%) 4.9% (0.0–12.5)	11 (17.5%) 25.4% (11.1–39.6)	31 (4.9%) 6.1% (3.5–8.7)	<0.001
Neither negative or positive	203 (42.2%) 37.5% (32.1–42.8)	54 (60.7%) 56.2% (41.0–71.4)	40 (63.5%) 49.9% (34.4–65.3)	297 (46.9%) 40.9% (36.1–45.8)	<0.001
Positive	263 (54.7%) 59.7% (54.2–65.1)	30 (33.7%) 38.9% (24.0–53.8)	12 (19%) 24.8% (11.0–38.6)	305 (48.2%) 53.0% (48.0–57.9)	<0.001

Note: * refers to weighted or adjusted percentage estimates from post-stratification of survey results.

## Data Availability

The data presented in this study are available on request from the corresponding author. The data are not publicly available due to data storage policy.
